# Sialylation regulates myofibroblast differentiation of human skin fibroblasts

**DOI:** 10.1186/s13287-017-0534-1

**Published:** 2017-04-18

**Authors:** Norihiko Sasaki, Yoko Itakura, Masashi Toyoda

**Affiliations:** Research Team for Geriatric Medicine (Vascular Medicine), Tokyo Metropolitan Institute of Gerontology, Sakaecho 35-2, Itabashi-ku, Tokyo, 173-0015 Japan

**Keywords:** Myofibroblast differentiation, Sialylation, Sialidase, CD44, Lipid rafts, Aging

## Abstract

**Background:**

Fibroblasts are key players in maintaining skin homeostasis and in orchestrating physiological tissue repair and skin regeneration. Dysfunctions in fibroblasts that occur with aging and the senescent process lead to the delayed healing observed in elderly people. The molecular mechanisms leading to fibroblast dysfunction during aging and the senescent process have not yet been clarified. Previously, changes in patterns of glycosylation were observed in fibroblasts in aging and the senescent process, but the effect of these changes on the function of fibroblasts has not been well documented. Here, we investigated whether changes in glycosylation during the process to senescence may have functional effects on fibroblasts.

**Methods:**

The changes in cell surface glycans on skin fibroblasts during the process to senescence were examined in early-passage (EP) and late-passage (LP) skin fibroblasts by fluorescence-activated cell sorting analysis using lectins. The contributors to the changes in cell surface glycans were examined by real-time polymerase chain reaction or Western blot analysis. The effects of changes in glycosylation on proliferation, migration, induction of cellular senescence, and myofibroblast differentiation induced by transforming growth factor (TGF)-β1 stimulation were examined in EP fibroblasts. The changes in glycosylation were performed by GalNAc-α-*O*-benzyl or sialidase treatment.

**Results:**

A decrease in sialylation of glycoproteins and an increase in sialidase NEU1 were observed in LP fibroblasts. The reduction of sialylation did not have any effect on proliferation, migration, or induction of cellular senescence. On the other hand, myofibroblast differentiation was inhibited by the reduction of sialylation, indicating that sialylation is important for myofibroblast differentiation. The localization of CD44 in lipid rafts, which is required for myofibroblast differentiation, was inhibited by the reduction of sialylation. Furthermore, reduced myofibroblast differentiation in LP fibroblasts was restored by a sialidase inhibitor.

**Conclusions:**

Desialylation of CD44 with increased sialidase during the process to senescence reduced the localization of CD44 in lipid rafts after TGF-β1 stimulation, leading to the inhibition of myofibroblast differentiation. Thus, regulation of sialylation may be an attractive strategy for the prevention and regenerative therapy of age-related skin diseases, cosmetic skin alterations, and chronic wounds caused by delayed healing in elderly people.

**Electronic supplementary material:**

The online version of this article (doi:10.1186/s13287-017-0534-1) contains supplementary material, which is available to authorized users.

## Background

With the dramatic expansion of the size of the elderly population (those over 65 years old), the risk of age-related pathologies, such as chronic wounds, and the associated clinical and socioeconomic burdens are increased. Therefore, research on the effects of aging on wound healing is a relevant topic. In response to injury, myofibroblasts, which are characterized by increased contractile ability and expression of α-smooth muscle actin (α-SMA) [1], differentiate from resident fibroblasts after proliferation and migration to the site of injury [1–3]. Myofibroblasts play important roles in the successful formation of granulation tissue and matrix remodeling in wound healing [4]. Therefore, myofibroblasts are considered key players in the delayed healing seen in elderly people. So far, it has been demonstrated that skin fibroblasts isolated from elderly people have impaired migration, premature senescence, impaired proliferative response, and defects in matrix generation as compared with skin fibroblasts from young people [5, 6]. Furthermore, it has been shown that aged fibroblasts resist myofibroblast differentiation [7]. However, the molecular mechanisms underlying these dysfunctions in fibroblasts with aging have not been clarified.

Carbohydrate antigens (also called glycans) expressed on the cell surface as components of glycoproteins change depending on the cellular state (developmental and pathological state), and contribute significantly to fundamental biological functions, such as cell differentiation, cell adhesion, cell-cell interaction, and regulation of signaling pathways [8]. Recent reports have demonstrated that cell surface glycans on fibroblasts change during aging and the senescent process; for instance, reduced sialylation on glycans was observed during cellular senescence in human lung fibroblasts [9–12]. Recently, our previous glycome analysis using lectin microarrays demonstrated changes in the total membrane glycosylation of glycoproteins in human skin fibroblasts with aging and the senescent process [13]. However, it is not known whether the changes in glycosylation have any effects on fibroblast function and senescence.

Here, we have investigated whether the changes in glycosylation during the process to senescence have functional effects on skin fibroblasts and have demonstrated novel mechanisms underlying age-related defects in myofibroblast differentiation.

## Methods

### Cell culture

TIG-3S cells (fetal-derived human skin fibroblasts) were purchased from Health Science Research Resources Bank (Osaka, Japan); the population doubling level (PDL) was 23. Cells were grown in Dulbecco’s modified Eagle medium (Wako Pure Chem. Ind. Co. Ltd., Osaka, Japan) containing 10% fetal bovine serum (Cell Culture Technologies, Gravesano, Switzerland), and penicillin-streptomycin (Gibco, Grand Island, NY, USA). Cells were passaged at a confluence of 80–90% and seeded at a density of 2500–5000 cells/cm^2^. All cells were studied at a confluence of 80–90%. PDLs were calculated at each passage using the following equation: *n* = (log2*X* – log2*Y*), where *n* = PDL, *X* = number of cells at the end of one passage, and *Y* = number of cells seeded at the beginning of one passage. In this study, fibroblasts with PDL 35–45 and PDL 73–78 were used as early-passage (EP) and late-passage (LP) fibroblasts, respectively. Cellular senescence occurred after PDL 80 as reported previously [13]. An in vitro aging model based on cellular senescence has previously been described and validated as a model of age-related alterations in human aortic smooth muscle cells, human vascular endothelial cells, and human fibroblasts [5, 6, 14–16]. Therefore, here we defined the LP fibroblasts as in vitro aged cells at the stage of pre-senescence, just before the process to cellular senescence. Doubling time was calculated by the formula PDL/time for increasing PDL. For myofibroblast differentiation, cells were cultured in serum-free medium containing 10 ng/ml human transforming growth factor (TGF)-β1 (Peprotech, Rocky Hill, NJ, USA). For depletion of sialic acid, cells were incubated with 2 mM GalNAc-α-*O*-benzyl (BGN; Sigma-Aldrich, St. Louis, MO, USA) or 100 U/ml sialidase from *C. perfringens* Type V (New England Biolabs, Ipswich, MA, USA) for at least 24 h and throughout the experiments. For inhibition of sialidase, cells were incubated with 2 mM 4-guanidino-2-deoxy-2,3-dehydro-*N*-acetylneuraminic acid (zanamivir; TCI, Tokyo, Japan) throughout the experiments.

### Senescence-associated β-galactosidase (SA-β-Gal) assay

SA-β-Gal activity was analyzed using a senescence detection kit (BioVision Inc., Milpitas, CA, USA) according to the manufacturer’s instructions. Briefly, cells were washed twice with phosphate-buffered saline (PBS), exposed to fixation solution for 10 min, and then incubated overnight in freshly prepared staining solution. After staining, cells were counterstained with 4',6-diamidino-2-phenylindole (DAPI), and then microscopic examination of four fields was performed. By counting the number of SA-β-Gal-positive cells based on their blue color and the total number of cells stained with DAPI using ImageJ software (National Institutes of Health, Bethesda, MD, USA), the percentage of SA-β-Gal-positive cells was calculated to estimate the percentage of senescent cells.

### Migration assay

The cells were cultured to confluence in 35-mm dishes. Next, a sterile 200-μl pipette tip was used to place a single wound across the diameter of each monolayer, and the medium was replaced to remove cell debris. The cells were then incubated for 8 h with medium containing serum. At 0 and 8 h, images of each monolayer were captured using a microscope coupled with a digital camera (Nikon, Tokyo, Japan). Cell migration into each wound after 8 h was compared with that observed in the same wounded monolayer at 0 h.

### Fluorescence-activated cell sorting (FACS) analysis

In general, trypsinization reduces the abundance of some cell surface antigens. Therefore, to avoid this effect, cells were harvested with Accutase® cell detachment solution (Gibco), and dissociated single cells were incubated with primary antibodies diluted in FACS buffer (0.5% (w/v) bovine serum albumin (BSA) and 0.1% (w/v) sodium azide in PBS) for 30 min on ice. After washing, the cell suspension was incubated with Alexa Fluor® 488-conjugated secondary antibodies (Molecular Probes, Eugene, OR, USA) diluted (1:400) in FACS buffer for 30 min on ice. Cell sorting and analysis were performed using a FACSAria™ Cell Sorter (Becton Dickinson, Franklin Lakes, NJ, USA). We used the following primary antibodies: monoclonal mouse anti-CD44 (dilution 1:100; R&D Systems Inc., Minneapolis, MN, USA), polyclonal rabbit anti-epidermal growth factor receptor (EGFR; dilution 1:100; GeneTex Inc., Irvine, CA, USA), and polyclonal rabbit anti-NEU1 (dilution 1:100; Thermo Fisher Scientific, Waltham, MA, USA). For lectin staining, FITC-conjugated *Wisteria floribunda* agglutinin (WFA; dilution 1:100; Vector Laboratories, Peterborough, UK), FITC-conjugated *peanut* agglutinin (PNA; dilution 1:100; J-Oil Mills, Tokyo, Japan), biotin-conjugated *Maackia amurensis* lectin II (MAL-II; dilution 1:100; Vector Laboratories), biotin-conjugated *Erythrina cristagalli* agglutinin (ECA; dilution 1:100; J-Oil Mills), and FITC-streptavidin (dilution 1:100; BioLegend, San Diego, CA, USA) were used. Mean fluorescence intensities (MFIs) were calculated by subtracting the intensities of the controls.

### Immunoblotting

For the analysis of TGF-β1 signaling, cell culture medium was replaced with serum-free medium for 24 h, and the cells were stimulated for 5 min with 10 ng/ml human TGF-β1. Cells were lysed with lysis buffer (50 mM Tris HCl pH 7.4, 150 mM NaCl, and 1% (v/v) Triton™ X-100) containing protease and phosphatase inhibitor cocktails (Roche, Indianapolis, IN, USA). For the ECA lectin pull-down assay, total cell lysates were incubated with biotin-conjugated ECA at 4 °C overnight. Then lysates were mixed with streptavidin-conjugated magnetic beads (Invitrogen, Carlsbad, CA, USA) and incubated for an additional 6 h. For immunoprecipitation (IP), cells were lysed with lysis buffer (50 mM Tris-HCl pH 7.4, 150 mM NaCl, 0.1% (v/v) SDS, 1% (v/v) sodium deoxycholate, and 1% (v/v) NP-40) containing protease and phosphatase inhibitor cocktails, and IPs were performed with the appropriate antibody and protein G magnetic beads (Veritas, Tokyo, Japan). Total lysates and precipitated proteins were separated by SDS-PAGE using a gel of the appropriate percentage and then transferred onto PVDF membranes (Merck Millipore, Billerica, MA, USA). After blocking, the membranes were incubated with the following primary antibodies: polyclonal rabbit anti-extracellular signal-regulated kinase 1/2 (ERK1/2; dilution 1:1000; Cell Signaling Technology, Danvers, MA, USA), polyclonal rabbit anti-phosphorylated ERK1/2 (pERK1/2; Thr183/185; dilution 1:1000; Cell Signaling Technology), monoclonal mouse anti-CD44 (dilution 1:1000), polyclonal rabbit anti-EGFR (dilution 1:1000; Abcam, Cambridge, UK), monoclonal mouse anti-α-SMA (dilution 1:1000; Abcam), polyclonal rabbit anti-NEU1 (dilution 1:1000), and monoclonal mouse anti-β-actin (dilution 1:10,000; Sigma-Aldrich). The membranes were then incubated with the appropriate peroxidase-conjugated secondary antibodies (dilution 1:30,000; Cell Signaling Technology), washed, and developed with ECL™ Prime reagents (GE Healthcare, Piscataway, NJ, USA).

### Immunostaining

Cells were fixed with 4% (w/v) paraformaldehyde and washed. Next, cells were permeabilized and blocked with PBS containing 0.2% (v/v) Triton™ X-100, 1% (w/v) BSA, and 5% (v/v) normal goat serum. After washing, cells were incubated with an anti-α-SMA antibody (dilution 1:100) at 4 °C overnight. After washing, cells were stained with an Alexa Fluor® 488-conjugated secondary antibody (dilution 1:400; Molecular Probes) and then counterstained with DAPI. For the observation of CD44, monosialotetrahexosylganglioside (GM1), and NEU1 on the cell surface, immunostaining was performed under non-permeabilized conditions using an anti-CD44 (dilution 1:100) or an anti-NEU1 primary antibody (dilution 1:100), an Alexa Fluor® 488-conjugated secondary antibody (dilution 1:400), and Alexa Fluor® 594-conjugated cholera toxin B subunit (dilution 1:200; Molecular Probes), which is known to bind to the raft-enriched ganglioside GM1. Immunofluorescence images were taken with a fluorescence microscope (Leica Microsystems, Wetzlar, Germany). Immunofluorescence images of colocalized CD44 with GM1 were taken with a confocal laser scanning microscope (Leica Microsystems). For the quantification of colocalized CD44 with GM1, the numbers of GM1-CD44 colocalized cells based on their yellow color and the total numbers of cells stained with DAPI at three fields (average total cell numbers at one field were 27 cells) were counted and then the percentage of GM1-CD44 colocalized cells was calculated.

### Real-time polymerase chain reaction (PCR)

Total RNA was isolated from cells using an RNeasy plus mini-kit (QIAGEN, Hilden, Germany) and subsequently reverse-transcribed using a ReverTra Ace® qPCR-RT Kit (Toyobo, Osaka, Japan). Real-time PCR was performed using a Power Sybr® Green kit (Applied Biosystems, Foster City, CA, USA) and a StepOnePlus™ real-time PCR system (Applied Biosystems). Primer sets for real-time PCR are listed in Table 1.

**Table 1:** List of primer sets for real-time polymerase chain reaction

Gene	Forward primer	Reverse primer
*NEU1*	TGTGACCTTCGACCCTGAGC	TCGCAGGGTCAGGTTCACTC
*NEU2*	AGTGGTCCACCTTTGCAGTG	ATGGCTGAGGAAGCAGAAGG
*NEU4*	TGCTGGTACCCGCCTACAC	CCGTGGTCATCGCTGTAGAA
*ST3Gal1*	ATACCCATCTACCGGCATCCT	AGTCCACCTCATCGCAGACAT
*ST3Gal3*	GCGTTCTTGCCAACAAGTCT	GTGATGCGCAGTGTCGTTT
*ST3Gal4*	GCAGAGAGCAAGGCCTCTAA	AGATCCTCACTCCCCTTGGT
*ST6Gal1*	GGGCAGGTGTGCTGTTGTG	TGCCTAGTTGGGAGGACTTCA
β*-actin*	GGTCATCACCATTGGCAATGAG	TACAGGTCTTTGCGGATGTCC

### Statistical analysis

Western blot images were densitometrically analyzed using ImageJ software (National Institutes of Health). Values are expressed as means ± standard deviation (SD) from three or four independent experiments. The Student’s *t* test for independent samples was used for statistical analysis.

## Results

### Sialylation of glycoproteins decreases in skin fibroblasts during the process to senescence

**Fig. 1:**
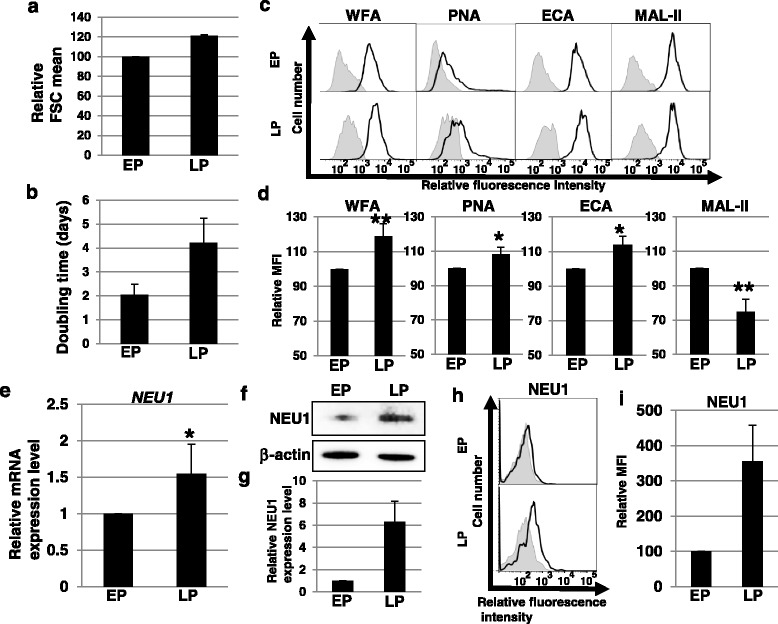
Sialylation of glycoproteins decreased during the process to senescence in skin fibroblasts. **a** Mean values of forward scatter (*FSC*) relative to early-passage (*EP*) fibroblasts are shown. Results are presented as means ± SD from three independent experiments. **b** The doubling time of the cells is shown. Results are presented as means ± SD from three independent experiments. **c**, **d** Cell surface glycans in EP and late-passage (*LP*) fibroblasts were analyzed by FACS using lectins. Three independent experiments were performed, and representative results are shown (**c**). Controls are presented in *gray*. Mean fluorescent intensities (*MFIs*) (after recalculation in relation to cell size as described in the Results section) relative to those of EP fibroblasts are shown (**d**). Results are presented as means ± SD from three or four independent experiments. *P < 0.05, **P < 0.02. **e** Real-time PCR analysis of *NEU1* was performed using cDNA derived from EP and LP fibroblasts. The results are shown after normalization to the values obtained for EP fibroblasts (value = 1). Results are presented as means ± SD from four independent experiments. *P < 0.05. **f**, **g** Western blot analysis of NEU1 was performed in EP and LP fibroblasts. The histogram (**g**) shows the mean densitometric analysis ± SD of NEU1 normalized to the loading control (β-actin). The values were obtained from three independent experiments. **h** Cell surface NEU1 in EP and LP fibroblasts was analyzed by FACS using anti-NEU1 antibody. Three independent experiments were performed, and representative results are shown. Controls are presented in *gray*. **i** MFIs relative to those of EP fibroblasts are shown. Results are presented as means ± SD from three independent experiments

Our previous report using lectin microarrays showed that changes in sialylation of glycoproteins occur in skin fibroblasts during aging and induction of senescence [13]. To confirm the changes in cell surface glycans on skin fibroblasts during the process to senescence, we performed FACS analysis using lectins (WFA, PNA, ECA, and MAL-II) in EP and LP skin fibroblasts. LP fibroblasts exhibited a larger cell size as measured by forward scatter (FSC) of FACS analysis and a lowered proliferative capacity (4.21 ± 1.02 vs. 2.04 ± 0.44 doubling times) (Fig. 1a and b), but the number of SA-β-gal-positive cells in LP fibroblasts was as low as in EP fibroblasts, indicating that senescence was not induced in LP fibroblasts (Additional file 1: Figure S1). Thus, LP fibroblasts were at the stage of the process to cellular senescence. FACS analysis revealed that binding of WFA (relative MFIs for EP vs LP, 100 vs 143.8 ± 8.7, *P <* 0.02), PNA (100 vs 131.1 ± 4.7, *P <* 0.02), and ECA (100 vs 137.8 ± 6.0, *P <* 0.02) significantly increased, whereas binding of MAL-II (100 vs 90.6 ± 8.7, *P =* 0.11) non-significantly decreased in LP fibroblasts as compared with EP fibroblasts (Fig. 1c). WFA (Galβ1-3GalNAc- and GalNAcβ1-4GlcNAc-), PNA (Galβ1-3GalNAc-), and ECA (Galβ1-4GlcNAc-) bind to the desialylated glycans, whereas MAL-II (Siaα2-3Gal-) binds to sialylated glycans, suggesting that sialylation of glycoproteins decreased per cell in LP fibroblasts. Furthermore, after recalculation of MFIs in relation to cell size (FSC is known to be proportional to cell diameter [17], and the cell size of LP fibroblasts was 1.21-times larger than that of EP fibroblasts, as shown in Fig. 1a), WFA, PNA, and ECA showed significantly increased, and MAL-II showed significantly decreased, binding per cell surface area in LP fibroblasts as compared to EP fibroblasts (Fig. 1d). Therefore, Fig. 1c and d indicates that sialylation of cell surface glycoproteins decreased during the process to senescence in skin fibroblasts. Sialylation is regulated by sialidases and sialyltransferases. Among four sialidases (NEU1, NEU2, NEU3, and NEU4), it is known that NEU3 is localized on the cell surface and its activity is specific for glycolipids. To elucidate the mechanisms contributing to the decreased sialylation of glycoproteins during the process to senescence, we analyzed the expression levels of three sialidases (NEU1, NEU2, and NEU4) and four sialyltransferases (ST3Gal1, ST3Gal3, ST3Gal4, and ST6Gal1). Real-time PCR analysis showed that the expression of *NEU1* was higher in LP fibroblasts than in EP fibroblasts (Fig. 1e). *NEU2* was not detected in either type. There were no significant differences in *NEU4* and sialyltransferase expression between EP fibroblasts and LP fibroblasts (Additional file 2: Figure S2). Furthermore, an increase in NEU1 protein levels was also observed in LP fibroblasts (Fig. 1f and g). Several reports have demonstrated that NEU1 localizes on the cell surface and modifies the sialylation of cell surface glycoproteins [18–21]. As shown in Fig. 1h and i and Figure S3 (Additional file 3), cell surface expression of NEU1 increased in LP fibroblasts. Taken together, these results demonstrate that sialylation of glycoproteins on the cell surface of skin fibroblasts was decreased during the process to senescence, presumably due to a cell surface increase in the expression of NEU1.

### Myofibroblast differentiation is inhibited by reduced sialylation with BGN

**Fig. 2:**
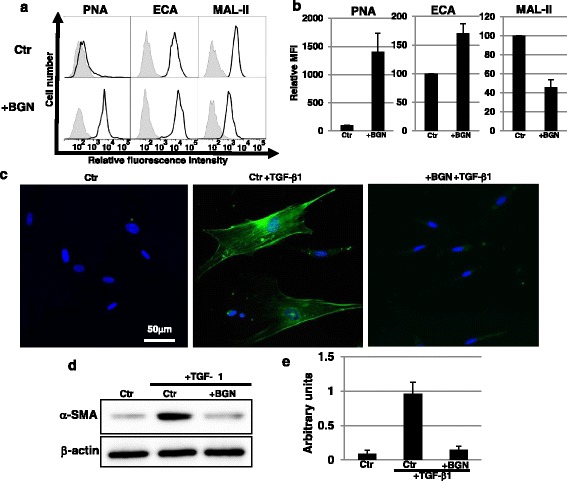
Myofibroblast differentiation was inhibited by the reduction of sialylation with BGN. **a**, **b** Cell surface glycans in control (*Ctr*; vehicle-treated EP fibroblasts in DMSO) and GalNAc-α-*O*-benzyl (*BGN*; 2 mM)-treated EP fibroblasts were analyzed by FACS using lectins. Three independent experiments were performed and representative results are shown (**a**). Controls are presented in *gray*. Mean fluorescent intensities (*MFIs*) relative to those of control cells are shown (**b**). Results are presented as means ± SD from three independent experiments. **c** Immunocytochemical staining was performed in control and BGN (2 mM)-treated EP fibroblasts 3 days after myofibroblast differentiation. Representative images are shown (α-SMA, *green*; DAPI, *blue*). **d**, **e** Western blot analysis of α-smooth muscle actin (α*-SMA*) was performed 3 days after myofibroblast differentiation in control and BGN (2 mM)-treated EP fibroblasts. The histogram (**e**) shows the mean densitometric analysis ± SD of α-SMA normalized to the loading control (β-actin). The values were obtained from three independent experiments. *TGF* transforming growth factor

Because it is not known whether changes in glycosylation during the process to senescence have an effect on the function of fibroblasts and on the induction of senescence, we examined the effect of decreased sialylation on EP fibroblasts using BGN. BGN inhibits glycosylation and causes changes in glycans, such as increasing Galβ1-3GalNAc structures and decreasing Siaα2-3Galβ1-3GalNAc structures [22]. Therefore, BGN treatment decreases sialylation to a similar extent as NEU1 overexpression. Treatment with BGN in EP fibroblasts increased binding of PNA and ECA, and decreased binding of MAL-II, indicating that glycoprotein sialylation was reduced, as observed in LP fibroblasts (Fig. 2a and b). BGN-treated EP fibroblasts did not show defects in proliferation and induction of senescence (Additional file 4: Figure S4a–c).

It is known that skin fibroblasts exhibit defects in several processes with aging, such as migration and myofibroblast differentiation [5–7]. First, we examined the effect of decreased sialylation using BGN on migration ability. As shown in Figure S4d (Additional file 4), migration was not different between control and BGN-treated EP fibroblasts, indicating that the decreased sialylation has no effect on migration ability. Next, we examined the effect of decreased sialylation on myofibroblast differentiation induced by TGF-β1, which is a key mediator of wound healing and is known to regulate myofibroblast differentiation [1, 23]. Immunocytochemical staining showed that TGF-β1 stimulation induced α-SMA-positive myofibroblast differentiation in control EP fibroblasts, but not in BGN-treated EP fibroblasts (Fig. 2c). Furthermore, Western blot analysis of α-SMA confirmed that myofibroblast differentiation was inhibited in BGN-treated EP fibroblasts (Fig. 2d and e). Thus, these results indicated that the reduction of sialylation by BGN inhibited myofibroblast differentiation induced by TGF-β1 stimulation.

### Raft localization of CD44 after TGF-β1 stimulation is inhibited by reduced sialylation with BGN

**Fig. 3:**
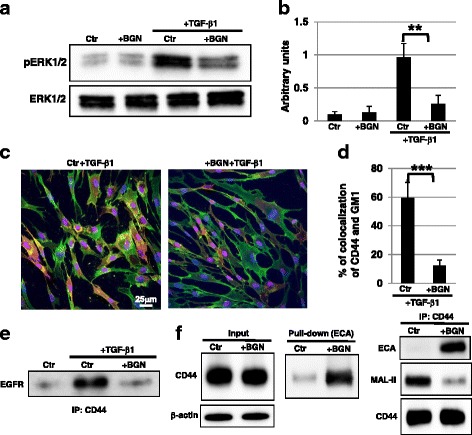
Raft localization of CD44 after TGF-β1 stimulation was inhibited by reduced sialylation with BGN. **a**, **b** Western blot analysis of phosphorylated extracellular signal-related kinase (*pERK*) was performed in control (*Ctr*; vehicle-treated EP fibroblasts in DMSO) and GalNAc-α-*O*-benzyl (*BGN*; 2 mM)-treated EP fibroblasts. The histogram (**b**) shows the mean densitometric analysis ± SD for the phosphorylated proteins normalized to the loading control. Values were obtained from three independent experiments. **P < 0.02. **c** Immunocytochemical staining was performed in control and BGN (2 mM)-treated EP fibroblasts after transforming growth factor (*TGF*)-β1 treatment. Representative images are shown (GM1, *red*; CD44, *green*; DAPI, *blue*; GM1 and CD44 colocalization, *yellow*). **d** The histogram shows the mean ± SD percentage of GM1-CD44 colocalized cells colored *yellow*, as shown in (**c**), from two independent experiments (total of six fields); ***﻿*P﻿* <0.01. **e** Immunoprecipitation (*IP*) of CD44 followed by immunoblotting of epidermal growth factor receptor (*EGFR*) was performed. Representative images are shown. **f** Total cell lysates from control and BGN-treated EP fibroblasts were pulled down with ECA. Immunoblotting of CD44 was performed on ECA-binding proteins. In addition, IP of CD44 followed by immunoblotting of ECA, MAL-II, and CD44 was performed. Representative images are shown

Because activation of the mitogen-activated protein kinase (MAPK/ERK) signaling by TGF-β1 stimulation is known to be required for myofibroblast differentiation [23], we examined whether the reduction of sialylation had an effect on TGF-β1–MAPK/ERK signaling. Western blot analysis showed that activation of the MAPK/ERK signaling by TGF-β1 stimulation was significantly reduced in BGN-treated EP fibroblasts (Fig. 3a and b). To elucidate the underlying mechanism causing the decreased MAPK/ERK signaling via the reduction of sialylation, we examined the expression of EGFR and CD44, which are involved in myofibroblast differentiation via TGF-β1–MAPK/ERK signaling [7]. The expression levels (total and cell surface) of EGFR and CD44 were not significantly changed after the reduction of sialylation by BGN treatment (Additional file 5: Figure S5). Recent reports have demonstrated that CD44 localization in lipid rafts after TGF-β1 stimulation is required for myofibroblast differentiation [23], and further sialylation on CD44 is required for lipid raft localization [24]. Next, we examined raft localization of CD44 in BGN-treated EP fibroblasts. Immunocytochemical staining showed that the ratio of colocalized CD44 with the raft marker GM1 in BGN-treated EP fibroblasts was lower than that in control EP fibroblasts, indicating that the localization of CD44 in lipid rafts was inhibited in BGN-treated EP fibroblasts (Fig. 3c and d). Furthermore, biochemical analysis showed that EGFR co-immunoprecipitation with CD44 was reduced in BGN-treated EP fibroblasts (Fig. 3e), indicating that interaction between EGFR and CD44 in the raft after TGF-β1 stimulation was inhibited by BGN treatment. We examined sialylation of CD44 after BGN treatment. ECA pull-down assays showed that the intensity of the CD44 band in BGN-treated EP fibroblasts was higher than in control EP fibroblasts, indicating desialylation on CD44 in BGN-treated EP fibroblasts (Fig. 3f). IP of the CD44 assay showed an increase in ECA and a decrease in MAL-II in BGN-treated EP fibroblasts, confirming that sialylation of CD44 was reduced by BGN treatment (Fig. 3f). Thus, these results indicate that raft localization of CD44 after TGF-β1 stimulation followed by interaction with EGFR was inhibited due to reduced CD44 sialylation by BGN treatment.

### Myofibroblast differentiation is inhibited by sialidase

**Fig. 4:**
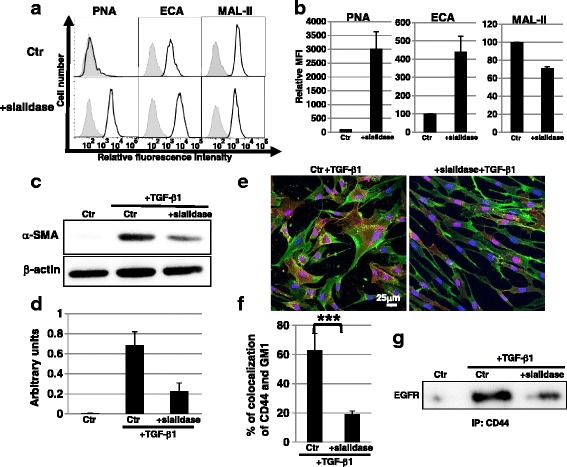
Myofibroblast differentiation was inhibited by sialidase. **a**, **b** Cell surface glycans in control (*Ctr*; non-treated EP fibroblasts) and 100 U/ml sialidase-treated EP fibroblasts were analyzed by FACS using lectins. Three independent experiments were performed and representative results are shown (**a**). Controls are presented in *gray*. Mean fluorescent intensities (*MFIs*) relative to those of control cells are shown (**b**). Results are presented as means ± SD from three independent experiments. **c**, **d** Western blot analysis of α-smooth muscle actin (α*-SMA*) was performed 3 days after myofibroblast differentiation in control and sialidase-treated EP fibroblasts. The histogram (**d**) shows the mean densitometric analysis ± SD of α-SMA normalized to the loading control (β-actin). The values were obtained from three independent experiments. **e** Immunocytochemical staining was performed in control and sialidase-treated EP fibroblasts after transforming growth factor (*TGF*)-β1 treatment. Representative images are shown (GM1, *red*; CD44, *green*; DAPI, *blue*; colocalization, *yellow*). **f** The histogram shows the mean ± SD percentage of GM1-CD44 colocalized cells colored *yellow*, as shown in (**e**), from two independent experiments (total of six fields); ***P <0.01. **g** Immunoprecipitation (*IP*) of CD44 followed by immunoblotting of epidermal growth factor receptor (*EGFR*) was performed. Representative images are shown

BGN treatment also affects the elongation of *O*-glycans. Therefore, to confirm the contribution of reduced sialylation, we further examined the effect of sialidase on EP fibroblasts. We used bacterial sialidase from *C. perfringens* Type V, which displays similar substrate specificity and extensive homology to NEU1 but not to the other three human sialidases [25]. The binding of PNA and ECA increased, whereas the binding of MAL-II decreased, after treatment of EP fibroblasts with sialidase, indicating that glycoprotein sialylation was reduced (Fig. 4a and b). The sialidase-treated EP fibroblasts did not show any defects in proliferation or migration, or in induction of senescence (Additional file 6: Figure S6). On the other hand, Western blot analysis of α-SMA demonstrated that the expression level of α-SMA was reduced in sialidase-treated EP fibroblasts even after TGF-β1 stimulation (Fig. 4c and d), indicating that myofibroblast differentiation was inhibited in sialidase-treated EP fibroblasts.

To clarify the mechanism underlying the reduced myofibroblast differentiation of sialidase-treated EP fibroblasts, first we examined the expression levels of EGFR and CD44. The expression levels (total and cell surface) of EGFR and CD44 did not significantly change after the sialylation was reduced by sialidase treatment (Additional file 7: Figure S7a and b). Next, we examined raft localization of CD44 in sialidase-treated EP fibroblasts. Immunocytochemical staining showed that the ratio of colocalized CD44 with the raft marker GM1 in sialidase-treated EP fibroblasts was lower than that in control EP fibroblasts, indicating that the localization of CD44 in lipid rafts was inhibited in sialidase-treated EP fibroblasts (Fig. 4e and f). Furthermore, biochemical analysis indicated that the interaction between EGFR and CD44 in lipid rafts after TGF-β1 stimulation was inhibited by sialidase treatment (Fig. 4g). IP of the CD44 assay using ECA and MAL-II demonstrated that sialylation of CD44 was reduced by sialidase treatment (Additional file 7: Figure S7c). Taken together, these results clearly indicate that sialic acid is required for the localization of CD44 in rafts after TGF-β1 stimulation followed by myofibroblast differentiation.

### Age-dependent reduction of myofibroblast differentiation is restored by a sialidase inhibitor

**Fig. 5:**
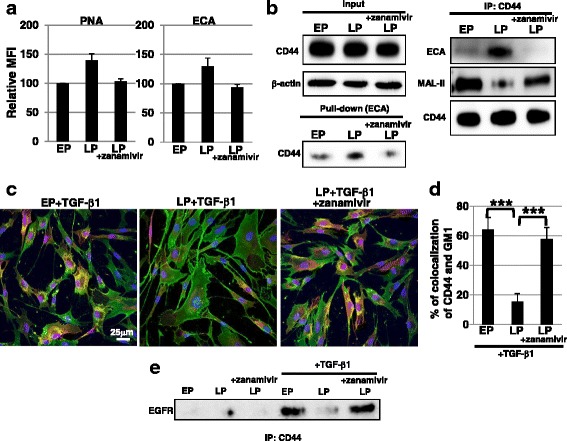
Defect in raft localization of CD44 during the process to senescence was restored by treatment with a sialidase inhibitor. **a** Cell surface glycans in early passage (*EP*), late passage (*LP*), and zanamivir (2 mM)-treated LP fibroblasts were analyzed by FACS using lectins. Mean fluorescent intensities (*MFIs*) relative to those of control cells are shown. Results are presented as means ± SD from three independent experiments. **b** Total cell lysates from EP, LP, and zanamivir-treated LP fibroblasts were pulled down with ECA. Immunoblotting of CD44 was performed on ECA-binding proteins (*left*). In addition, immunoprecipitation (*IP*) of CD44, followed by immunoblotting of ECA, MAL-II, and CD44, was performed (*right*). Representative images are shown. **c** Immunocytochemical staining was performed in EP, LP, and zanamivir-treated LP fibroblasts after transforming growth factor (*TGF*)-β1 treatment. Representative images are shown (GM1, *red*; CD44, *green*; DAPI, *blue*; colocalization, *yellow*). **d** The histogram shows the mean ± SD percentage of GM1-CD44 colocalized cells colored *yellow*, as shown in (**c**), from two independent experiments (total of six fields); ***P <0.01. **e** IP of CD44 followed by immunoblotting of epidermal growth factor receptor (*EGFR*) was performed. Representative images are shown

As demonstrated above, myofibroblast differentiation by TGF-β1 stimulation was inhibited by the reduction of sialylation. It is also known that aging fibroblasts resist myofibroblast differentiation due to a reduction in EGFR and hyaluronic acid (HA) levels [7]. Furthermore, HA synthesized via HAS2 after TGF-β1 stimulation contributes to relocalization of CD44 to lipid rafts [23]. In our study, there were no differences in cell surface levels of EGFR and CD44 between EP and LP fibroblasts (Additional file 8: Figure S8a). Furthermore, there were no differences in *HAS2* expression between EP and LP fibroblasts even after TGF-β1 stimulation, indicating that the synthesis of HA in LP fibroblasts was not reduced (Additional file 8: Figure S8b). Therefore, we hypothesized that the reduction of CD44 sialylation during the process to senescence would inhibit myofibroblast differentiation in LP fibroblasts. As shown in Fig. 1, the reduction of sialylation in LP fibroblasts correlated with a cell surface increase in sialidase NEU1 expression. Thus, to clarify that reduced sialylation contributes to inhibition of myofibroblast differentiation in LP fibroblasts, we examined the effect of a sialidase inhibitor on LP fibroblasts. FACS analysis showed that treatment with zanamivir, which is an inhibitor of mammalian sialidase including NEU1 [20, 26], reduced the binding of PNA and ECA to LP fibroblasts to the same levels as observed in EP fibroblasts (Fig. 5a). These results indicate that an increase in sialidase expression during senescence reduced sialylation in LP fibroblasts. An ECA pull-down assay showed that the band of CD44 was more intense in LP fibroblasts than in EP fibroblasts, indicating the desialylation of CD44 in LP fibroblasts (Fig. 5b, left). After zanamivir treatment, desialylation of CD44 in LP fibroblasts was restored, as shown in Fig. 5b. Furthermore, IP CD44 assay using ECA and MAL-II confirmed that sialylation of CD44 was reduced in LP fibroblasts, but was restored by zanamivir treatment (Fig. 5b, right). Next, we examined raft localization of CD44 after TGF-β1 stimulation in LP fibroblasts. Immunocytochemical staining showed that the localization of CD44 in lipid rafts was reduced in LP fibroblasts, but zanamivir treatment restored raft localization of CD44 in these cells (Fig. 5c and d). Furthermore, biochemical analysis showed that the reduced interaction of CD44 and EGFR in LP fibroblasts was restored by zanamivir treatment (Fig. 5e). Thus, these results indicate that reduced CD44 sialylation affects raft localization of CD44 after TGF-β1 stimulation followed by interaction with EGFR in LP fibroblasts.

**Fig. 6:**
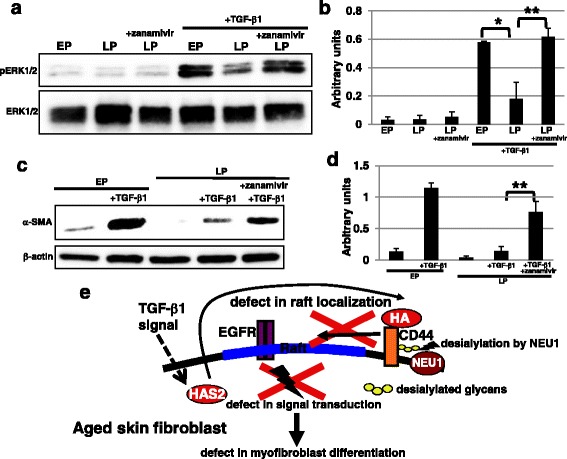
Age-dependent reduction of myofibroblast differentiation was restored by a sialidase inhibitor. **a**, **b** Western blot analysis of phosphorylated extracellular signal-related kinase (*pERK*) was performed in early passage (*EP*), late passage (*LP*), and zanamivir (2 mM)-treated LP fibroblasts. The histogram (**b**) shows mean densitometric readings ± SD for the phosphorylated proteins normalized to the loading controls. Values were obtained from three independent experiments. *P < 0.05, **P < 0.02. **c**, **d** Western blot analysis for α-smooth muscle actin (α*-SMA*) was performed 3 days after myofibroblast differentiation in EP, LP, and zanamivir-treated LP fibroblasts. The histogram (**d**) shows mean densitometric readings ± SD of α-SMA normalized to the loading control (β-actin). The values were obtained from three independent experiments. **P < 0.02. **e** Schematic representation of myofibroblast differentiation regulated by CD44 sialylation. Hyaluronic acid (*HA*) synthesized via HAS2 after transforming growth factor (*TGF*)-β1 stimulation contributes to the relocalization of CD44 to lipid rafts; the association between epidermal growth factor receptor (*EGFR*) and CD44 then activates MAPK/ERK signaling required for myofibroblast differentiation [23]. In aged fibroblasts, increased expression of the sialidase NEU1 contributes to the desialylation of CD44, which is unable to relocalize to lipid rafts even after TGF-β1 stimulation. Therefore, CD44 cannot bind anymore to the EGFR, leading to the inhibition of myofibroblast differentiation

Therefore, we next examined whether the treatment with the sialidase inhibitor zanamivir could restore myofibroblast differentiation in LP fibroblasts. As shown in Fig. 6a and b, impaired MAPK/ERK signaling by TGF-β1 stimulation in LP fibroblasts was restored by zanamivir treatment. Furthermore, Western blot analysis of α-SMA showed that myofibroblast differentiation in LP fibroblasts was restored after zanamivir treatment (Fig. 6c and d). Collectively, these results indicate that the reduction in CD44 sialylation via increased sialidase NEU1 expression inhibits myofibroblast differentiation in LP fibroblasts.

## Discussion

Myofibroblasts, which differentiate from migrated fibroblasts, play pivotal roles in maintaining skin homeostasis and orchestrating physiological tissue repair and skin regeneration. Therefore, dysfunctions in fibroblasts such as decreased proliferation, migration, and myofibroblast differentiation lead to repair defects or to injuries with damaged and/or cosmetic skin alterations such as wrinkle development. The molecular mechanisms of fibroblast dysfunctions during aging have not yet been elucidated. In this study, we proposed a novel mechanism for myofibroblast differentiation (Fig. 6e). HA is synthesized via HAS2 after TGF-β1 stimulation contributing to CD44 relocalization to lipid rafts; the association between EGFR and CD44 then activates MAPK/ERK signaling required for myofibroblast differentiation in non-aged fibroblasts [23]. In in vitro aged fibroblasts, increased NEU1 sialidase expression contributes to desialylation of CD44, which is unable to associate with raft-localized EGFR even after TGF-β1 stimulation, affecting MAPK/ERK signaling and inhibiting myofibroblast differentiation. Thus, we propose that the reduction of sialylation in fibroblasts with aging is a risk factor for age-related pathologies, including chronic wounds in elderly people.

Sialic acids, with relatively strong electronegative charges, on glycans contribute to many cellular functions including migration and proliferation; hence, changes in sialylation levels have been considered to be functionally involved in developmental and pathological states [8, 27]. Recent reports using lectin microarrays have demonstrated changes in sialylation of glycoproteins during senescence in human mesenchymal stem cells and human skin fibroblasts [13, 28]. Here, we show that sialylation of glycoproteins in human skin fibroblasts was decreased during the process to senescence via increased sialidase NEU1 expression. Therefore, changes in sialylation may contribute to dysfunctions in in vitro aged skin fibroblasts, such as premature senescence and decreased migration and proliferation [5, 6], although the molecular mechanisms underlying those dysfunctions with aging have not been clarified. Next, in our study, we examined how reduced sialylation might affect skin fibroblast function. Our results demonstrated that the reduction of sialylation with BGN or sialidase treatment has no effect on cellular senescence, proliferation, or migration (Additional file 4: Figure S4 and Additional file 6: Figure S6). It is well known that glycans other than sialic acid play functional roles in proliferation and migration in several cell types including fibroblasts [8, 29, 30], but their role in aging has not been elucidated. In our previous study, we demonstrated changes in glycosylation of glycoproteins, such as *N*- and *O*-glycans, in human skin fibroblasts during aging and the senescent process [13]. Therefore, glycans other than sialic acid may have functional roles during those processes. Further studies are required to clarify whether changes in glycosylation with aging have an effect on skin fibroblasts.

In our study, myofibroblast differentiation was inhibited in LP fibroblasts, even though cell surface levels of EGFR and CD44 did not change in serum-free culture conditions or normal culture conditions in non-confluent cells (Additional file 8: Figure S8a). Furthermore, there were no differences in *HAS2* expression between EP and LP fibroblasts after TGF-β1 stimulation (Additional file 8: Figure S8b). It has been demonstrated that reduction of EGFR and HA levels during senescence in confluent dermal fibroblasts inhibits myofibroblast differentiation in serum-free conditions [7]. Here, we show that CD44 sialylation affects myofibroblast differentiation. It is known that the ability of myofibroblast to differentiate is cell context-dependent [31]. Therefore, the differences between our results and past reports may be due to the type of fibroblasts and cell culture conditions used. We also show that reduction of CD44 sialylation during the process to senescence affected raft localization of CD44 after TGF-β1 stimulation. Because CD44 sialylation negatively regulates HA binding [32], reduced mobility of CD44 due to the binding between desialylated CD44 and HA may inhibit CD44 relocalization to lipid rafts. Furthermore, raft localization of CD44 may depend on the interactions between sialic acid and raft-resident proteins, and sialic acid-binding lectins may be good candidates. At the moment, it is unknown what kinds of sialylated structures on CD44 are critical for the function of CD44, but it is considered that sialyl T antigen (Siaα2-3Galβ1-3GalNAc-) is included on CD44 from the results using sialidase or sialidase inhibitor (Fig. 5b and Additional file 7: Figure S7c). It has been demonstrated that sialyl T antigen in the raft-localized protein, such as integrin β4, is important for integrin β4-mediated signaling [33]. Thus, sialyl T antigen may be functional on raft-localized CD44 for the signal transduction in lipid rafts. Regardless, further studies are required to clarify the mechanisms underlying the inhibition of myofibroblast differentiation with aging due to the affected mobility of desialylated CD44 and to identify the candidate partners of CD44 in lipid rafts and functional sialylated structures on CD44.

Sialidase NEU1 has been shown to be involved in epithelial to mesenchymal transition in pancreatic cancer due to activation of matrix metalloproteinase 9-EGFR signaling after reduction of EGFR sialylation [34, 35]. NEU1 is involved in the activation of dendritic and macrophage cells by desialylation of Toll-like receptor 4, and in the recruitment of leukocytes by removal of sialyl residues from the activation epitopes on the adhesion molecules of endothelial cells [36]. Hence, an increase in NEU1 may cause chronic inflammation, resulting in the initiation and progression of several diseases such as cancers, rheumatoid arthritis, and atherosclerosis. Therefore, an increase in NEU1 expression with aging can be a risk factor for such diseases in elderly people. In our study, we show an increase in NEU1 expression in skin fibroblasts during the process to senescence. NEU1 has substrate specificity for α2-3 sialyl linkage [37]. Indeed, in our study (Fig. 1c and d and Fig. 5b) and our previous report [13], a decrease in α2-3 sialyl linkage with aging was indicated from the decrease in the binding of MAL-II. Therefore, NEU1 and also NEU1-recognizing glycans, such as α2-3 sialyl linkages, may be attractive targets for the prevention and therapy of age-related skin diseases, cosmetic skin alterations, and chronic wounds caused by delayed healing in elderly people.

## Conclusions

In this study, we showed that raft localization of CD44 after TGF-β1 stimulation was reduced by desialylation with increased sialidase during the process to senescence, leading to the inhibition of myofibroblast differentiation in skin fibroblasts. Our results suggest that regulation of sialylation in fibroblasts may be an attractive strategy for the prevention and regenerative therapy of age-related skin diseases, cosmetic skin alterations, and chronic wounds caused by delayed healing in elderly people.

## Additional files

Additional file 1: Figure S1. Senescence was not induced in LP fibroblasts. EP fibroblasts and LP fibroblasts were stained for SA-β-Gal activity. Representative images of staining for SA-β-Gal and DAPI are shown. (PPTX 3118 kb)

Additional file 2: Figure S2. The expression levels of *NEU4* and sialyltransferases did not differ between EP and LP fibroblasts. Real-time PCR analysis of *NEU4* and sialyltransferases was performed using cDNA derived from EP and LP fibroblasts. The results are shown after normalization to the values obtained for EP fibroblasts (value = 1). Results are presented as means ± standard deviation (SD) from three independent experiments. (PPTX 73 kb)

Additional file 3: Figure S3. NEU1 expression increases on the cell surface of LP fibroblasts. Immunocytochemical staining was performed in EP and LP fibroblasts under non-permeabilzed and permeabilized conditions. Representative images are shown (NEU1, *green*; DAPI, *blue*). (PPTX 2462 kb)

Additional file 4: Figure S4. Reduction of sialylation by GalNAc-α-*O*-benzyl (BGN) treatment had no effects on proliferation, migration, or induction of cellular senescence. **a** The growth rate of EP fibroblasts 3 days after culture with or without BGN is shown. The results are shown after normalization to the values obtained for control cells (value = 1). Results are presented as means ± SD from three independent experiments. **b**, **c** EP fibroblasts 3 weeks after culture with or without BGN were stained for SA-β-Gal activity, and SA-β-Gal-positive cells were quantified as a percentage of total cells. Representative images of staining for SA-β-Gal and DAPI are shown (**b**). Results are presented as means ± SD from four fields (**c**). **d** A wound was performed on confluent cultures of control and BGN-treated EP fibroblasts, which were then incubated for 8 h. Representative phase-contrast images from three independent experiments are shown. Control (Ctr): vehicle-treated EP fibroblasts (DMSO). (PPTX 4766 kb)

Additional file 5: Figure S5. Reduction of sialylation by GalNAc-α-*O*-benzyl (BGN) treatment did not affect EGFR and CD44 expression levels. **a**, **b** Western blot analysis of EGFR or CD44 was performed on total cell lysates of control and BGN-treated EP fibroblasts. The histogram (**b**) shows the mean densitometric analysis ± SD of EGFR or CD44 normalized to the loading control (β-actin). The results are shown after normalization to the values obtained for control cells (value = 1). The values were obtained from three independent experiments. **c** FACS analysis of cell surface EGFR or CD44 was performed in control and BGN-treated EP fibroblasts. MFIs relative to the control cells are shown (value = 100). Results are presented as means ± SD from three independent experiments. Control (Ctr): vehicle-treated EP fibroblasts (DMSO). (PPTX 399 kb)

Additional file 6: Figure S6. Reduction of sialylation by sialidase treatment had no effects on proliferation, migration, or induction of cellular senescence. **a** The growth rate of EP fibroblasts 3 days after culture with or without sialidase is shown. The results are shown after normalization to the values obtained for control cells (value = 1). Results are presented as means ± SD from three independent experiments. **b** EP fibroblasts 3 weeks after culture with or without sialidase were stained for SA-β-Gal activity. Representative images of staining for SA-β-Gal and DAPI are shown. **c** A wound was performed on confluent cultures of control and sialidase-treated EP fibroblasts, which were then incubated for 24 h. Representative phase-contrast images are shown. Control (Ctr): non-treated EP fibroblasts. (PPTX 3893 kb)

Additional file 7: Figure S7. Reduction of sialylation by sialidase treatment did not affect EGFR or CD44 expression levels. **a** Western blot analysis of EGFR or CD44 was performed on total cell lysates of control and sialidase-treated EP fibroblasts. Representative images are shown. **b** FACS analysis of cell surface EGFR or CD44 was performed in control and sialidase-treated EP fibroblasts. MFIs relative to the control cells are shown (value = 100). Results are presented as means ± SD from three independent experiments. **c** Immunoprecipitation (IP) of CD44 followed by immunoblotting of ECA, MAL-II, and CD44 was performed in control and sialidase-treated EP fibroblasts. Representative images are shown. Control (Ctr): non-treated EP fibroblasts. (PPTX 606 kb)

Additional file 8: Figure S8. Cell surface expression of EGFR and CD44, and *HAS2* expression did not decrease in LP fibroblasts. **a** Cell surface expression of EGFR and CD44 in EP and LP fibroblasts did not change after culture in serum-free medium. FACS analysis of cell surface EGFR or CD44 was performed in EP and LP fibroblasts after 0, 24, and 48 h of culture without serum. MFIs relative to EP fibroblasts are shown at each point of culture (value = 100). Results are presented as means ± standard deviation from three independent experiments. **b** Real-time PCR analysis of *HAS2* was performed using cDNA derived from EP and LP fibroblasts after 0 or 6 h of culture with TGF-β1. The results are shown after normalization to the values obtained for non-treated EP fibroblasts (value = 1). Results are presented as means ± SD from three independent experiments. (PPTX 99 kb)

## Abbreviations

BGN: GalNAc-α-*O*-benzyl
BSA: Bovine serum albumin
DAPI: 4',6-Diamidino-2-phenylindole
ECA: *Erythrina cristagalli* agglutinin
EGFR: Epidermal growth factor receptor
EP: Early-passage
ERK: Extracellular signal-regulated kinase
FACS: Fluorescence-activated cell sorting
FSC: Forward scatter
GM1: Monosialotetrahexosylganglioside
HA: Hyaluronic acid
IP: Immunoprecipitation
LP: Late-passage
MAL-II: *Maackia amurensis* lectin II
MAPK: Mitogen-activated protein kinase
MFI: Mean fluorescence intensity
PBS: Phosphate-buffered saline
PCR: Polymerase chain reaction
PDL: Population doubling level
PNA: *Peanut* agglutinin
SA-β-Gal: Senescence-associated β-galactosidase
SD: Standard deviation
TGF: Transforming growth factor
WFA: *Wisteria floribunda* agglutinin
α-SMA: α-smooth muscle actin
