# Supplementary figures and images for: Sialylation regulates myofibroblast differentiation of human skin fibroblasts
Source: Stem Cell Res Ther. 2017 Apr 18;8:81. doi: 10.1186/s13287-017-0534-1 (PMC5395757; doi:10.1186/s13287-017-0534-1)

## Slide 1
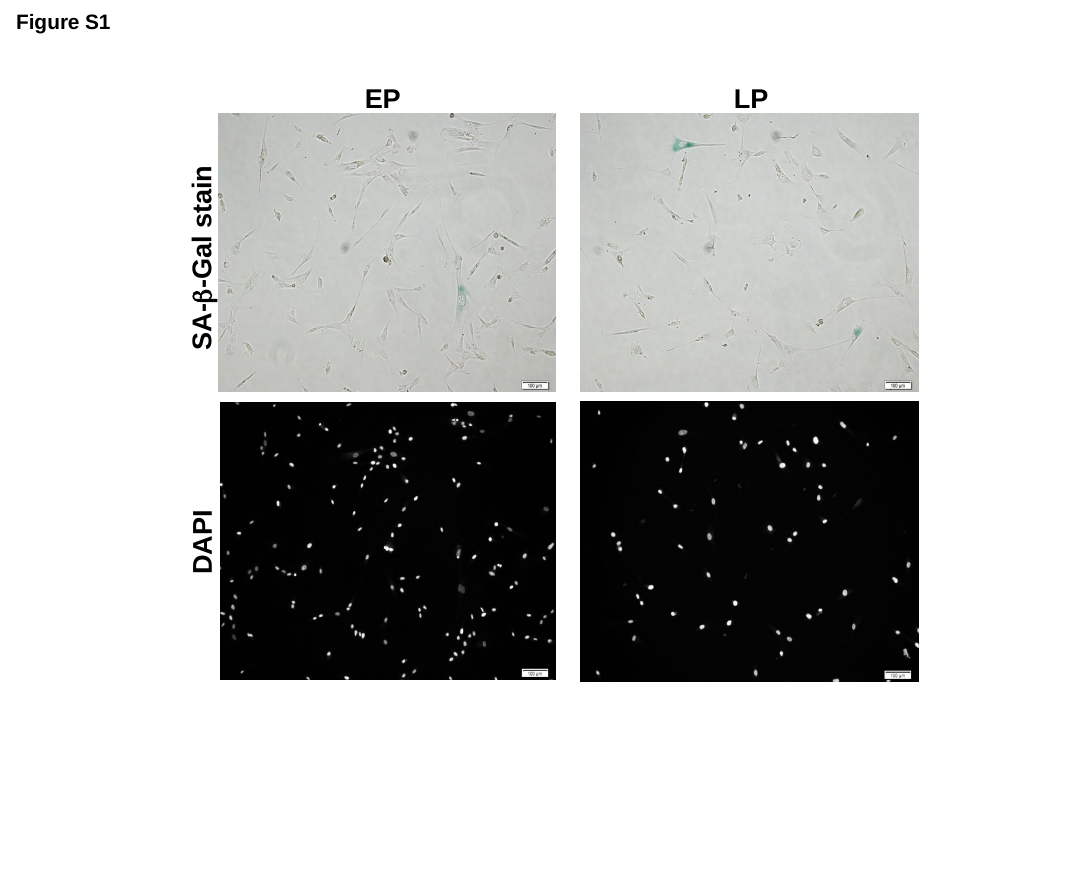

Figure S1
EP
LP
 SA-b-Gal stain
DAPI

Supplement: Supplementary file 1 — Senescence was not induced in LP fibroblasts. EP fibroblasts and LP fibroblasts were stained for SA-β-Gal activity. Representative images of staining for SA-β-Gal and DAPI are shown. (PPTX 3118 kb) [file 13287_2017_534_MOESM1_ESM.pptx]

## Slide 1
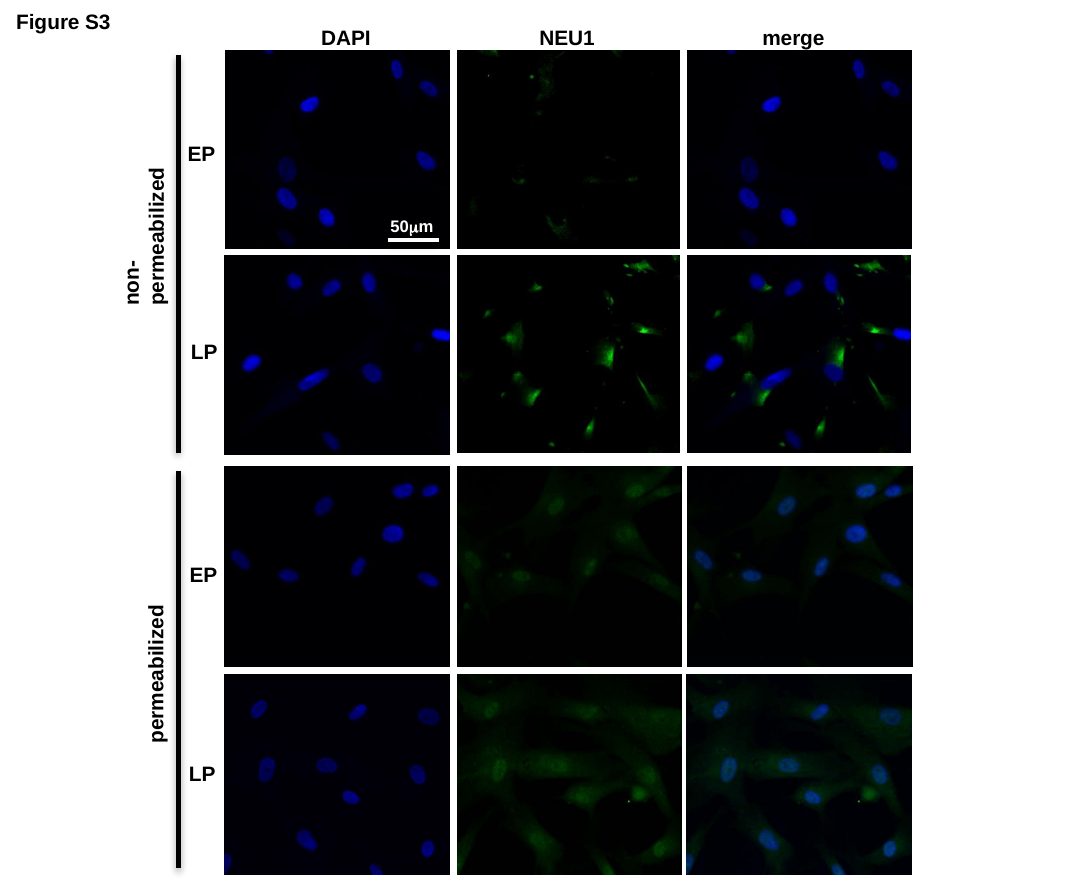

Figure S3
DAPI
NEU1
merge
EP
non-
permeabilized
50mm
LP
EP
permeabilized
LP

Supplement: Supplementary file 3 — NEU1 expression increases on the cell surface of LP fibroblasts. Immunocytochemical staining was performed in EP and LP fibroblasts under non-permeabilzed and permeabilized conditions. Representative images are shown (NEU1, green; DAPI, blue). (PPTX 2462 kb) [file 13287_2017_534_MOESM3_ESM.pptx]
