# Supplementary material for: Sialylation regulates myofibroblast differentiation of human skin fibroblasts
Source: Stem Cell Res Ther. 2017 Apr 18;8:81. doi: 10.1186/s13287-017-0534-1 (PMC5395757; doi:10.1186/s13287-017-0534-1)
Supplement: Supplementary file 2 — The expression levels of NEU4 and sialyltransferases did not differ between EP and LP fibroblasts. Real-time PCR analysis of NEU4 and sialyltransferases was performed using cDNA derived from EP and LP fibroblasts. The results are shown after normalization to the values obtained for EP fibroblasts (value = 1). Results are presented as means ± standard deviation (SD) from three independent experiments. (PPTX 73 kb) [file 13287_2017_534_MOESM2_ESM.pptx]

## Slide 1
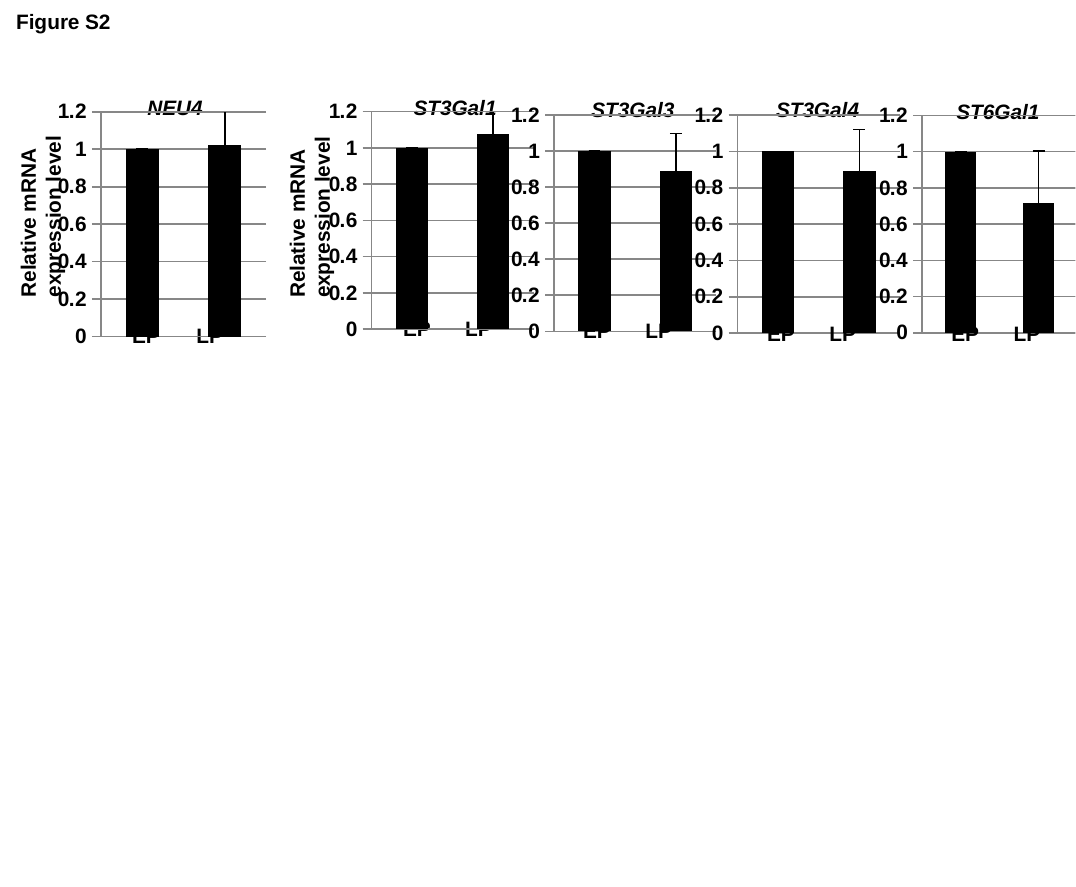

Figure S2
NEU4
ST3Gal1
ST3Gal3
ST3Gal4
ST6Gal1
### Chart
| Category | |
|---|---|
### Chart
| Category | |
|---|---|
### Chart
| Category | |
|---|---|
### Chart
| Category | |
|---|---|
### Chart
| Category | |
|---|---|Relative mRNA
expression level
Relative mRNA
expression level
EP
LP
EP
LP
EP
LP
EP
LP
EP
LP
