# Supplementary material for: Sialylation regulates myofibroblast differentiation of human skin fibroblasts
Source: Stem Cell Res Ther. 2017 Apr 18;8:81. doi: 10.1186/s13287-017-0534-1 (PMC5395757; doi:10.1186/s13287-017-0534-1)
Supplement: Supplementary file 4 — Reduction of sialylation by GalNAc-α-O-benzyl (BGN) treatment had no effects on proliferation, migration, or induction of cellular senescence. a The growth rate of EP fibroblasts 3 days after culture with or without BGN is shown. The results are shown after normalization to the values obtained for control cells (value = 1). Results are presented as means ± SD from three independent experiments. b, c EP fibroblasts 3 weeks after culture with or without BGN were stained for SA-β-Gal activity, and SA-β-Gal-positive cells were quantified as a percentage of total cells. Representative images of staining for SA-β-Gal and DAPI are shown (b). Results are presented as means ± SD from four fields (c). d A wound was performed on confluent cultures of control and BGN-treated EP fibroblasts, which were then incubated for 8 h. Representative phase-contrast images from three independent experiments are shown. Control (Ctr): vehicle-treated EP fibroblasts (DMSO). (PPTX 4766 kb) [file 13287_2017_534_MOESM4_ESM.pptx]

## Slide 1
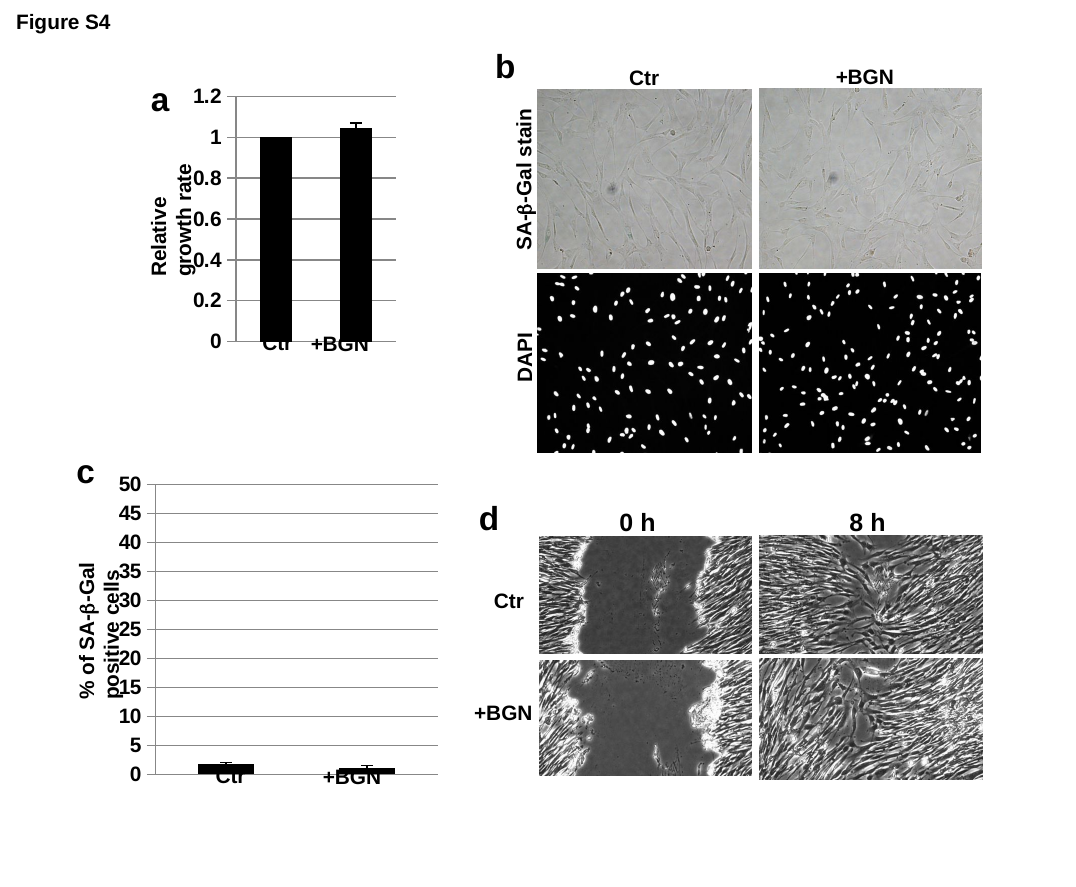

Figure S4
b
+BGN
Ctr
a
### Chart
| Category | |
|---|---|
 SA-b-Gal stain
Relative growth rate
Ctr
+BGN
DAPI
c
### Chart
| Category | |
|---|---|d
0 h
8 h
Ctr
% of SA-b-Gal
positive cells
+BGN
Ctr
+BGN
