# Supplementary material for: Sialylation regulates myofibroblast differentiation of human skin fibroblasts
Source: Stem Cell Res Ther. 2017 Apr 18;8:81. doi: 10.1186/s13287-017-0534-1 (PMC5395757; doi:10.1186/s13287-017-0534-1)
Supplement: Supplementary file 5 — Reduction of sialylation by GalNAc-α-O-benzyl (BGN) treatment did not affect EGFR and CD44 expression levels. a, b Western blot analysis of EGFR or CD44 was performed on total cell lysates of control and BGN-treated EP fibroblasts. The histogram (b) shows the mean densitometric analysis ± SD of EGFR or CD44 normalized to the loading control (β-actin). The results are shown after normalization to the values obtained for control cells (value = 1). The values were obtained from three independent experiments. c FACS analysis of cell surface EGFR or CD44 was performed in control and BGN-treated EP fibroblasts. MFIs relative to the control cells are shown (value = 100). Results are presented as means ± SD from three independent experiments. Control (Ctr): vehicle-treated EP fibroblasts (DMSO). (PPTX 399 kb) [file 13287_2017_534_MOESM5_ESM.pptx]

## Slide 1
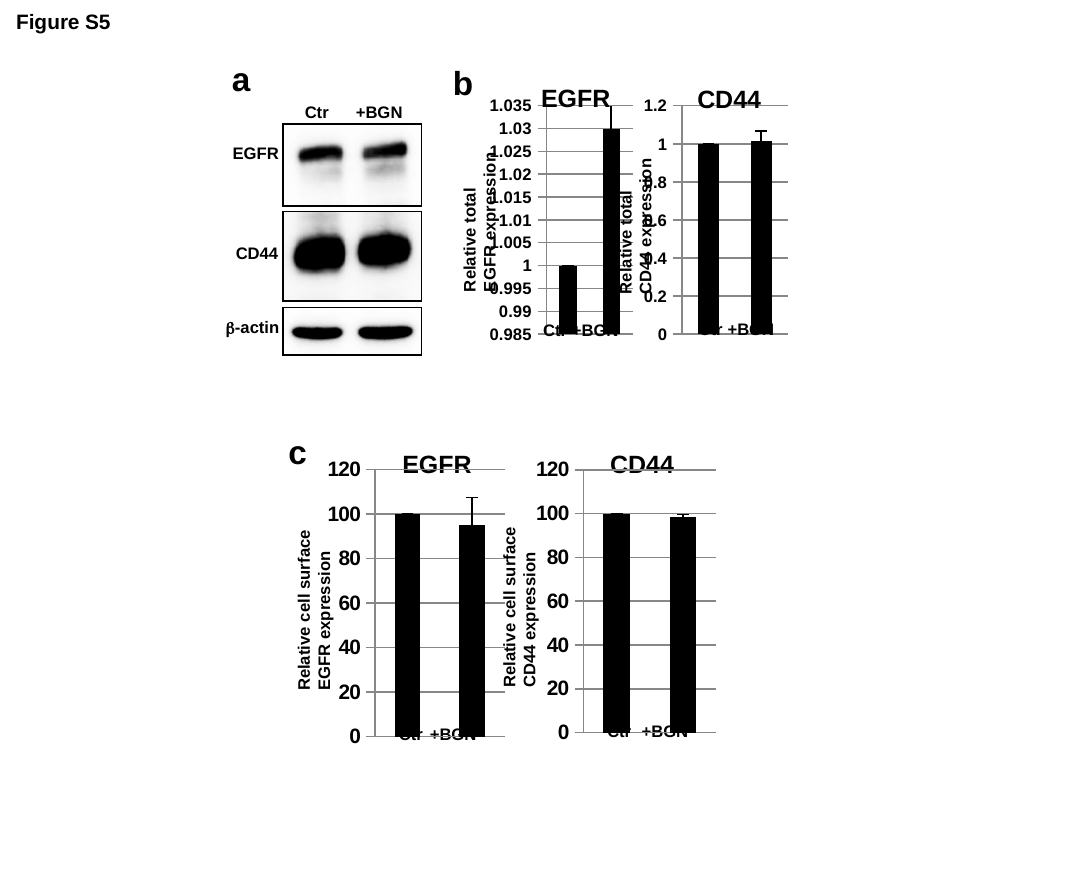

Figure S5
a
b
EGFR
CD44
### Chart
| Category | |
|---|---|
### Chart
| Category | |
|---|---|Ctr
+BGN
EGFR
Relative total
CD44 expression
Relative total
EGFR expression
CD44
b-actin
Ctr
+BGN
Ctr
+BGN
c
EGFR
CD44
### Chart
| Category | |
|---|---|
### Chart
| Category | |
|---|---|Relative cell surface
CD44 expression
Relative cell surface
EGFR expression
Ctr
+BGN
Ctr
+BGN
