# Supplementary material for: Sialylation regulates myofibroblast differentiation of human skin fibroblasts
Source: Stem Cell Res Ther. 2017 Apr 18;8:81. doi: 10.1186/s13287-017-0534-1 (PMC5395757; doi:10.1186/s13287-017-0534-1)
Supplement: Supplementary file 6 — Reduction of sialylation by sialidase treatment had no effects on proliferation, migration, or induction of cellular senescence. a The growth rate of EP fibroblasts 3 days after culture with or without sialidase is shown. The results are shown after normalization to the values obtained for control cells (value = 1). Results are presented as means ± SD from three independent experiments. b EP fibroblasts 3 weeks after culture with or without sialidase were stained for SA-β-Gal activity. Representative images of staining for SA-β-Gal and DAPI are shown. c A wound was performed on confluent cultures of control and sialidase-treated EP fibroblasts, which were then incubated for 24 h. Representative phase-contrast images are shown. Control (Ctr): non-treated EP fibroblasts. (PPTX 3893 kb) [file 13287_2017_534_MOESM6_ESM.pptx]

## Slide 1
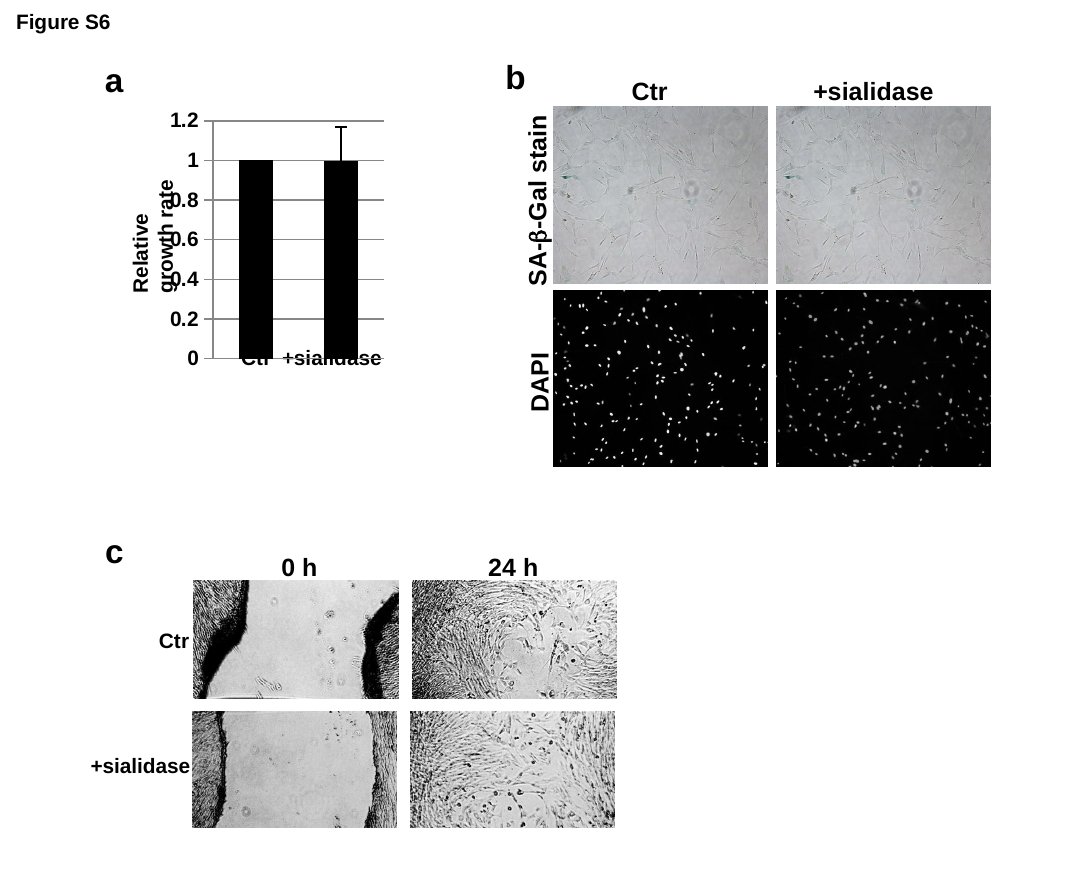

Figure S6
b
a
Ctr
+sialidase
### Chart
| Category | |
|---|---|
 SA-b-Gal stain
Relative growth rate
Ctr
+sialidase
DAPI
c
0 h
24 h
Ctr
+sialidase
