# Supplementary material for: Sialylation regulates myofibroblast differentiation of human skin fibroblasts
Source: Stem Cell Res Ther. 2017 Apr 18;8:81. doi: 10.1186/s13287-017-0534-1 (PMC5395757; doi:10.1186/s13287-017-0534-1)
Supplement: Supplementary file 7 — Reduction of sialylation by sialidase treatment did not affect EGFR or CD44 expression levels. a Western blot analysis of EGFR or CD44 was performed on total cell lysates of control and sialidase-treated EP fibroblasts. Representative images are shown. b FACS analysis of cell surface EGFR or CD44 was performed in control and sialidase-treated EP fibroblasts. MFIs relative to the control cells are shown (value = 100). Results are presented as means ± SD from three independent experiments. c Immunoprecipitation (IP) of CD44 followed by immunoblotting of ECA, MAL-II, and CD44 was performed in control and sialidase-treated EP fibroblasts. Representative images are shown. Control (Ctr): non-treated EP fibroblasts. (PPTX 606 kb) [file 13287_2017_534_MOESM7_ESM.pptx]

## Slide 1
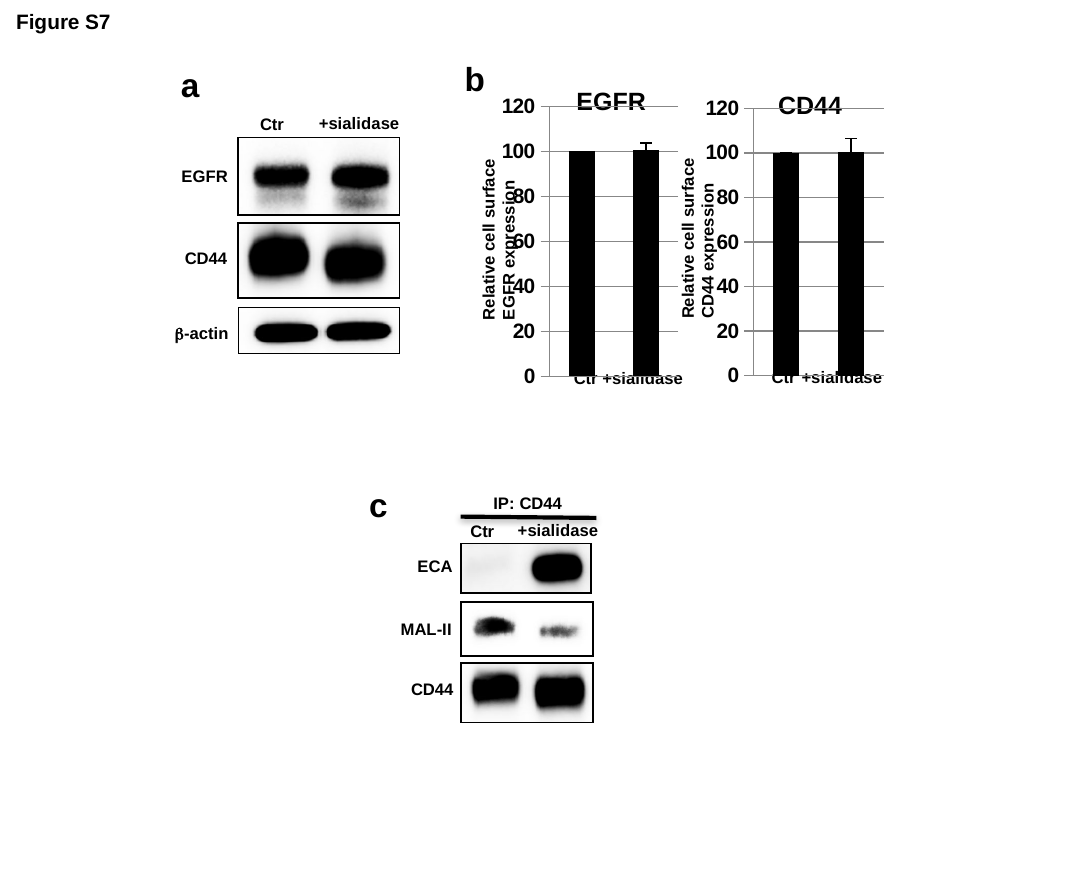

Figure S7
b
a
EGFR
CD44
### Chart
| Category | |
|---|---|
### Chart
| Category | |
|---|---|+sialidase
Ctr
EGFR
Relative cell surface
CD44 expression
Relative cell surface
EGFR expression
CD44
b-actin
Ctr
+sialidase
Ctr
+sialidase
c
IP: CD44
+sialidase
Ctr
ECA
MAL-II
CD44
