# Supplementary material for: Sialylation regulates myofibroblast differentiation of human skin fibroblasts
Source: Stem Cell Res Ther. 2017 Apr 18;8:81. doi: 10.1186/s13287-017-0534-1 (PMC5395757; doi:10.1186/s13287-017-0534-1)
Supplement: Supplementary file 8 — Cell surface expression of EGFR and CD44, and HAS2 expression did not decrease in LP fibroblasts. a Cell surface expression of EGFR and CD44 in EP and LP fibroblasts did not change after culture in serum-free medium. FACS analysis of cell surface EGFR or CD44 was performed in EP and LP fibroblasts after 0, 24, and 48 h of culture without serum. MFIs relative to EP fibroblasts are shown at each point of culture (value = 100). Results are presented as means ± standard deviation from three independent experiments. b Real-time PCR analysis of HAS2 was performed using cDNA derived from EP and LP fibroblasts after 0 or 6 h of culture with TGF-β1. The results are shown after normalization to the values obtained for non-treated EP fibroblasts (value = 1). Results are presented as means ± SD from three independent experiments. (PPTX 99 kb) [file 13287_2017_534_MOESM8_ESM.pptx]

## Slide 1
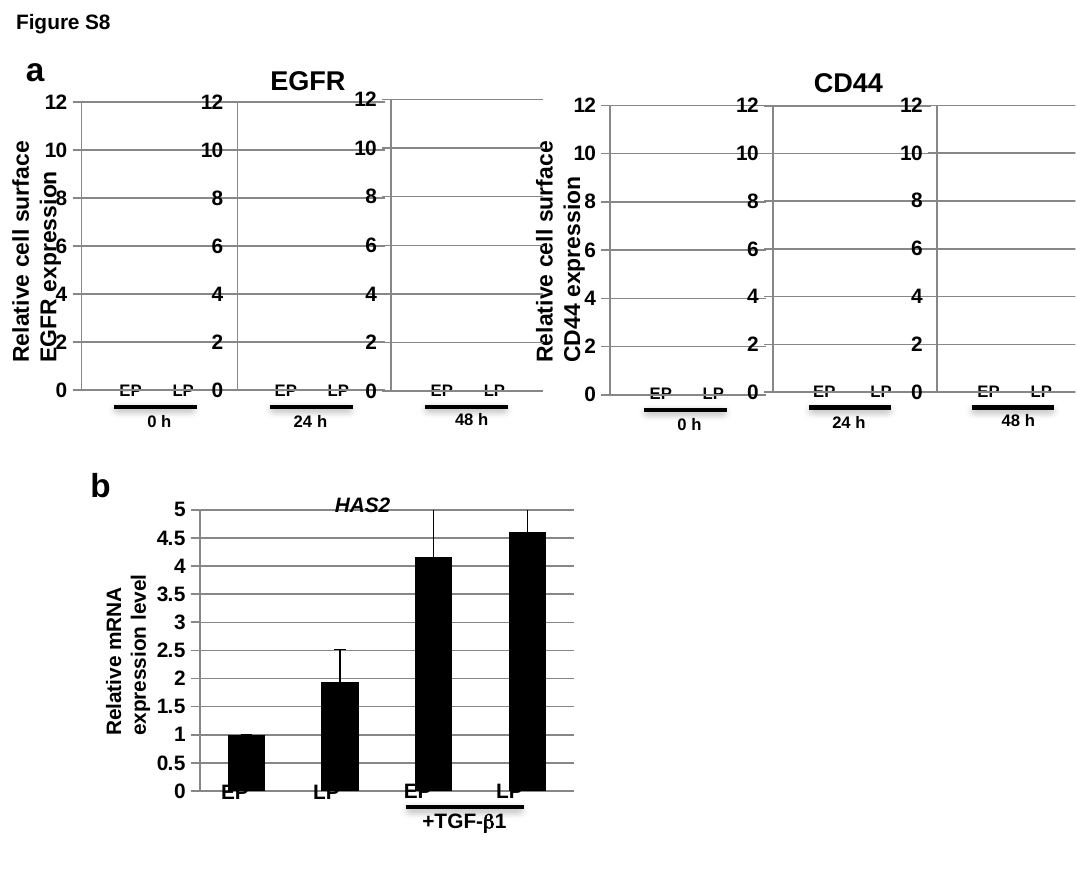

Figure S8
a
EGFR
CD44
### Chart
| Category | |
|---|---|
### Chart
| Category | |
|---|---|
### Chart
| Category | |
|---|---|
### Chart
| Category | |
|---|---|
### Chart
| Category | |
|---|---|
### Chart
| Category | |
|---|---|Relative cell surface
EGFR expression
Relative cell surface
CD44 expression
EP
LP
EP
LP
EP
LP
EP
LP
EP
LP
EP
LP
48 h
48 h
0 h
24 h
24 h
0 h
b
HAS2
### Chart
| Category | |
|---|---|Relative mRNA
expression level
EP
LP
EP
LP
+TGF-b1
